# Infection prevention preparedness and practices for female sterilization services within primary care facilities in Northern India

**DOI:** 10.1186/s12913-019-4778-6

**Published:** 2019-12-31

**Authors:** Abhishek Kumar, Abhishek Gautam, Arnab Dey, Ruhi Saith, Uttamacharya, Pranita Achyut, Vandana Gautam, Dinesh Agarwal, Amit Chakraverty, Arupendra Mozumdar, Kumudha Aruldas, Ravi Verma, Priya Nanda, Suneeta Krishnan, Niranjan Saggurti

**Affiliations:** 1 Population Council, B 86, Defense Colony, New Delhi, 110024 India; 2 International Center for Research on Women, New Delhi, India; 3 Sambodhi Research and Communications Private Limited, Noida, Uttar Pradesh India; 4 Oxford Policy Management, New Delhi, India; 5 IPE Global, New Delhi, India; 6 Bill and Melinda Gates Foundation, New Delhi, India

## Abstract

**Background:**

In 2014, 16 women died following female sterilization operations in Bilaspur, a district in central India. In addition to those 16 deaths, 70 women were hospitalized for critical conditions (Sharma, Lancet 384,2014). Although the government of India’s guidelines for female sterilization mandate infection prevention practices, little is known about the extent of infection prevention preparedness and practice during sterilization procedures that are part of the country’s primary health care services. This study assesses facility readiness for infection prevention and adherence to infection prevention practices during female sterilization procedures in rural northern India.

**Method:**

The data for this study were collected in 2016–2017 as part of a family planning quality of care survey in selected public health facilities in Bihar (*n* = 100), and public (*n* = 120) and private health facilities (*n* = 97) in Uttar Pradesh. Descriptive analysis examined the extent of facility readiness for infection prevention (availability of handwashing facilities, new or sterilized gloves, antiseptic lotion, and equipment for sterilization). Correlation and multivariate statistical methods were used to examine the role of facility readiness and provider behaviors on infection prevention practices during female sterilization.

**Result:**

Across the three health sectors, 62% of facilities featured all four infection prevention components. Sterilized equipment was lacking in all three health sectors. In facilities with all four components, provider adherence to infection prevention practices occurred in only 68% of female sterilization procedures. In Bihar, 76% of public health facilities evinced all four components of infection prevention, and in those facilities provider’s adherence to infection prevention practices was almost universal. In Uttar Pradesh, where only 55% of public health facilities had all four components, provider adherence to infection prevention practices occurred in only 43% of female sterilization procedures.

**Conclusion:**

The findings suggest that facility preparedness for infection prevention does play an important role in provider adherence to infection prevention practices. This phenomenon is not universal, however. Not all doctors from facilities prepared for infection prevention adhere to the practices, highlighting the need to change provider attitudes. Unprepared facilities need to procure required equipment and supplies to ensure the universal practice of infection prevention.

## Background

Adherence to infection prevention practices – before, during, and after surgery or invasive procedures – is essential to prevent patient morbidity as well as mortality [1–3]. Inadequate infection prevention practices can lead to surgical site infections including sepsis, tetanus, and other infections such as HIV and AIDS or Hepatitis C [4–8]. Like other health care surgeries, clinical family planning services – female sterilization and Intrauterine Contraceptive Device (IUCD) insertion – require adherence to infection prevention practices to prevent ensuing complications. Adherence to infection prevention practices not only avoids post-procedural complications but enhances contraceptive method continuation as well as attracts new users [9–16].

Female sterilization is the most common contraceptive method used by couples in India [17]. The incidence of 16 deaths in 2014 following female sterilizations at medical camps in Bilaspur in central India drew both national and international attention to India’s quality of care during sterilization procedures [18]. Official data presented to India’s Parliament in 2014 reported more than 360 deaths across the country following female sterilization procedures between April 2010 and March 2013 [19]. Although the exact causes of those deaths were not determined, these sterilization tragedies were reported as a consequence of surgeries under unsafe and unhygienic conditions [19, 20]. Infections, due to septic conditions at surgery, after female sterilization have been evident for decades and are leading causes of death associated with female sterilization in Asia and Latin America [21]. A study from Bangladesh found that anesthetic complication is the most frequent cause of sterilization-attributable deaths, followed by infection, and hemorrhage [6]. Those three leading causes of death were also associated with laparoscopic sterilization deaths in the United States [22]. These studies suggest that maintenance of aseptic conditions during female sterilization is still a critical challenge across developed and developing countries.

Adherence to infection prevention practice is a desired technical competence included in the six elements of the quality of care framework in family planning, proposed by Bruce and Jain in 1990 [23]. According to the World Health Organization’s infection prevention control and measure guidelines, hand hygiene, use of personal protective equipment, sterilization, and medical device de-contamination are essential components of health care infection prevention [24]. Hand washing and scrubbing before clinical procedures is the single most effective means of preventing infection transmission in hospitals [25]. Similarly, use of discrete sets of sterile surgical gloves for every procedure is an essential measure for preventing surgical site infection, by creating a physical barrier against micro-organisms [26, 27]. Meanwhile, equipment sterilization is effective in eliminating all micro-organisms including endospores [28], and cleaning of incision area with antiseptic solution prior to incision reduces risk of infection at the surgical site [29]. Adherence to all of these essential practices depends upon factors including health care providers’ knowledge of these practices, their training, and most importantly, infrastructure and availability of equipment and supplies to facilitate compliance during the procedure [25, 30].

Although adherence to infection prevention per standard operating procedures for family planning dictates appropriate care to avoid complications and prevent deaths, little is known about such practices in India. The present study assesses facility readiness for infection prevention and adherence to infection prevention practices during female sterilization in two northern states of India.

## Methods

This study was part of a large-scale baseline assessment of quality of care in family planning in health facilities in two northern states of India – Bihar and Uttar Pradesh (UP). These two states have high fertility rates, low family planning use, and high unmet need for contraception compared to major states in India as well as the national average. Contribution of female sterilization to modern contraceptive prevalence rate was 89% in Bihar and 54% in UP [31, 32].

This study utilizes quality of care data collected in facility audits and observations of female sterilization in public health facilities in Bihar and both public and private health facilities in Uttar Pradesh from June 2016 to February 2017. In all three of those health sectors, facilities were selected using stratified random sampling strategies. Sector-specific selection of health facilities for the survey is described below.

### Sample selection for Bihar public facilities

A total sample of 100 facilities allowed an inter-temporal minimum detectable effect of 13.1% for facility indicators in the state. The sampling frame comprised a total of 580 public health facilities (all facilities upto Primary Health Center level) in the state of Bihar. All of the facilities were divided into three strata – District Hospital (DH), Community Health Center (CHC), and Primary Health Center (PHC). The number of facilities selected from each stratum was determined by the relative proportion of each type from the list of intervention and non-intervention facilities. Within each stratum, health facilities were selected using probability proportional to size (PPS) sampling; facilities that conducted more mini-lap procedures in the previous year had a higher probability of being selected in the sample.

### Sample selection for UP public facilities

In Uttar Pradesh, public health facilities of a district were stratified into only two groups – DH and CHC. Of the total sample of facilities, 50% were selected from the state-specific program’s priority districts, while the other 50% were from non-priority districts. The priority districts are the districts with low utilisation of reproductive and child health services (institutional delivery, childhood immunization, and family planning), high maternal and infant mortality, and high total fertility rate; whereas non-priority districts have comparatively better healthcare coverage and outcomes [33]. DHs and CHCs in each district were selected using PPS sampling, based on numbers of sterilization procedures during the preceding year. Sterilization procedure numbers were determined to provide sufficient numbers of new family planning users. Thus, 100 public health facilities with high client loads were selected initially; later, 20 more facilities were included to achieve the required sample size, for a total of 120 public health facilities included in Uttar Pradesh.

### Sample selection for UP private (social franchise) clinics

First, 10 districts with clinics managed by Population Services International (PSI), and 17 by Hindustan Latex Family Planning Promotion Trust (HLFPPT), were listed in Uttar Pradesh. PSI districts were stratified into two groups, and HLFPPT into three, by geography. Within each stratum, districts were arranged by modern contraceptive prevalence rate (based upon Annual Health Survey, 2012–13), and seven districts from each stratum were selected using systematic random sampling. All social franchise clinics of these networks with caseloads of 20 or more were included in the study, resulting in 97 facilities represented: 60 in the PSI network and 37 from HLFPPT.

### Data collection procedures

This study’s data were drawn from assessments of infection prevention preparedness and practices, through facility audits and direct observations of female sterilization procedures, respectively. The facility audit checklist collected data on the presence of trained staff at facility; basic infrastructure (i.e. availability of potable water, electricity, functional toilets, and shaded waiting area); availability of drugs, equipment, and supplies for clinical family planning services; availability of commodities for non-clinical family planning methods; and equipment and materials for infection prevention measures. The observation checklist collected data on measures for standard operating procedures of female sterilization recommended by the Government of India. In Bihar, researchers observed the initial four or five sterilization cases, whereas in UP, the cases for observations happened throughout the day. In Bihar, female sterilization services are provided on fixed days, where medical doctors or surgeons arrive at health facilities in the late afternoon (usually coming from district hospitals or nearby health facilities); this arrangement demanded data collectors to limit the observation to initial few sterilization procedures. It is important to mention that this study is part of a large-scale baseline assessment of quality of care in family planning and have data on other aspects too – apart from the facility audits and the direct observation, but we only analyzed those data which are relevant for this study. Some data collected from other instruments are published in another article [34].

### Measures

This study considered two aspects of infection prevention:

#### Facility readiness for infection prevention

Facility readiness for infection prevention was assessed using four components, such as availability of – handwashing facilities in operation theatre; sterilized surgical gloves; spirit or povidone-Iodine solution (antiseptic solution); and a room or area with autoclave or boiler, surgical drum, and cidex for sterilizing equipment. While assessing facility readiness for infection prevention, the proportion of facilities with staff trained in infection prevention, responsible for equipment sterilization, was also evaluated. All these components are related to facility readiness for infection prevention.

#### Adherence to infection prevention practices during the female sterilization procedure

Four infection prevention practices – washing or scrubbing hands before procedure, use of sterilized gloves for every procedure, use of high-level disinfectant (HLD) equipment for every procedure, and painting incision area with antiseptic solution – were considered as key infection prevention practices. In the analysis, these practices were first analyzed separately, and then a composite variable was created, coded as 1 if all four practices were adhered to, and 0 otherwise.

Covariates adjusted in the analysis for examining infection prevention practices were type of facility (Bihar public, UP public, UP private), availability of staff trained in infection prevention responsible for equipment sterilization (available, not available), availability of at least one provider trained in female sterilization (available, not available), and facility preparedness for infection prevention (yes, no).

### Data analysis

Univariate analyses assessed facility readiness for infection prevention and level of infection prevention practices. Bivariate analyses were carried out to examine the association between infection prevention practices and facility preparedness. Pearson correlation analysis was applied to examine the direction of association between infection prevention preparedness and practice, with facility readiness and infection prevention practice scores used. Facility readiness scores were generated by adding all four components of facility readiness for infection prevention. Since each variable was coded as 1 if a component was available in a facility, and 0 otherwise, scores ranged from 0 to 4, where 0 indicates no component available in a facility and 4 indicates all four components were available. Similar scores for infection prevention were computed. Multivariate analysiss is used to examine the determinants of infection prevention practices, with the outcome variable adherence to infection prevention practice – whether or not all four practices (handwashing, use of new or sterilized gloves, use of sterilized equipment, and use of antiseptic lotion to clean the incision area) were employed during clinical procedures. The outcome was dichotomized based on the four infection prevention practices mentioned and coded as 1 if all four practices were adhered to, and 0 otherwise. Since the outcome variable was dichotomous, logistic regression analysis was used, after adjusting for few selected characteristics and results are presented as adjusted odds ratios (AOR) along with 95% of confidence interval (CI). All analyses presented hereafter were carried out using STATA 13.0 [35].

## Results

### Facility readiness for infection prevention

In aggregate, 62% of health facilities in the two states featured all four components of infection prevention (availability of – handwashing facilities, gloves, antiseptic lotions, and sterilization room or area with necessary items for instrument sterilization, Table 1). Availability of gloves and antiseptic solution were almost universal, but a sterilization room or area with the necessary items were found in only 72% of facilities. Facility readiness varied among the three health sectors: 81% of public facilities in Bihar had all four components, but in Uttar Pradesh, only 53% of public and 63% of private health facilities had all four components. Availability of a sterilization room equipped with necessary items was low in both UP’s public (58%) and private (73%) facilities. Only 46% of all facilities in both states had a person responsible for sterilizing equipment is trained in infection prevention.

**Table 1:** Percentage of facilities ready to provide infection prevention measures and infection prevention practices in family planning services in the three health sectors

Components of facility readiness for infection prevention	Bihar public	Uttar Pradesh public	Uttar Pradesh private	Total
Infection prevention components (%)				
Having handwashing facility in operation theater	88.2	84.2	95.9	89.0
Availability of gloves	100.0	92.5	97.4	96.1
Availability of spirit/povidone-iodine solution	84.5	97.5	96.1	93.2
Sterilization room/area having necessary items	100.0	58.3	73.2	72.4
*All four above*	*81.1*	*51.7*	*63.2*	*61.5*
Staff who is responsible for sterilizing equipment is trained in infection prevention	17.2	52.5	63.9	45.5
Number of facilities	100	120	97	317
Infection prevention practices (%)				
Washed hands before every observed case	86.8	97.0	83.6	91.8
Used new/sterilized gloves for every observed case	97.9	100.0	96.4	98.9
Used antiseptic solution to clean insertion area	86.2	80.5	70.9	82.2
Used sterilized equipment for every observed case	96.6	55.8	72.7	74.0
*All four procedures practiced*	*73.3*	*45.5*	*45.5*	*57.1*
Number of sterilization procedure observed	342	400	55	797

### Adherence to infection prevention practices during female sterilization

All four infection prevention practices were followed in only 57% of female sterilization procedures in all three health sectors (Table 1). New or sterilized gloves were used in almost every procedure, hands were scrubbed during 92% of procedures, and antiseptic solution was used in 82% of procedures, while sterilized equipment was used in just 74% of procedures. Adherence to infection prevention practices varied by health sector – 73% in public health facilities of Bihar, and 46% each in public and private health facilities of Uttar Praesh. Of the four prevention practices, use of sterilized equipment and antiseptic lotion to clean the incision area were lowest in both the public and private health facilities of Uttar Pradesh.

Among facilities prepared for infection prevention, only 68% followed all four infection prevention practices (Table 2). Hand washing or scrubbing, using new or sterile gloves, and applying antiseptic lotion at the incision site were generally practiced (88–99%) in facilities prepared for that infection prevention component; but sterilized equipment was used in only 79% of cases, even when the autoclave (or) boiler was available. Moreover, in facilities with available staff trained in infection prevention, sterilized equipment was used in only 51% of cases. In Bihar, 76% of facilities were fully prepared for infection prevention, with practice nearly always coinciding in the observed initial four or five cases (89%). In UP’s public sector, however, 55% of facilities were fully ready for infection prevention, but in only 43% of cases (observed throughout the day), all four practices were followed. A similar result was observed in private health facilities of Uttar Pradesh. Use of sterilized equipment was limited in Uttar Pradesh, even when facilities had sterilizing equipment.

**Table 2:** Infection prevention readiness and practices adopted during female sterilization in the three sectors

	Sample size: number of facilities/observed cases	Fully ready four^b^ (%)	Wash/scrub hands (%)	Gloves (%)	Antiseptic solution (%)	Sterilized equipment (%)
Total						
Facility readiness for IP^a^	212	63.0	86.0	95.0	92.0	81.0
IP practiced where facilities are ready	797	68.0	94.0	99.0	88.0	80.0
IP practiced in facilities having trained staff		51.0	94.0	99.0	78.0	67.0
Bihar public						
Facility readiness for IP	82	76.2	86.8	100.0	100.0	84.5
IP practiced where facilities are ready	342	88.7	92.1	97.9	100.0	97.2
IP practiced in facilities having trained staff		64.1	82.8	98.4	78.1	90.6
UP public						
Facility readiness for IP	120	54.7	84.2	92.5	97.5	65.8
IP practiced where facilities are ready	400	42.6	97.1	100.0	80.3	52.3
IP practices in facilities having trained staff		46.3	99.0	100.0	78.6	57.7
UP private						
Facility readiness for IP	19	31.6	84.2	68.4	73.7	68.4
IP practiced where facilities are ready	55	52.6	100.0	100.0	78.1	89.2
IP practices in facilities having trained staff		54.4	87.0	95.7	73.9	76.1

^a^IP: Infection prevention

^b^Fully ready includes scrub hands, use of gloves, use of sterilized equipment, and use of antiseptic lotion

### Association between facility readiness for infection prevention and practice

Pearson correlation analysis examined the association between facility readiness for infection prevention (availability of – handwashing facilities, gloves, antiseptic lotion, and sterilized equipment) and practices during female sterilization. A score was generated for readiness and for practices by summing variables considered. When all health facilities are considered, there is a significant positive correlation (correlation coefficient [CC] = 0.299; *p* < 0.001) between facility readiness and infection prevention practices during female sterilization (Table 3). This correlation was found to be significant in public health facilities in Bihar (CC 0.712; *p* < 0.001) but not in Uttar Pradesh.

**Table 3:** Correlation coefficient showing the association between infection prevention readiness and practices for female sterilization across health sectors

Facilities	Female sterilization
Total	0.298^*^
Bihar public facilities	0.712^*^
Uttar Pradesh public facilities	0.023
Uttar Pradesh private facilities	−0.103

^*^p < 0.05

Availability of all four components in facilities was a significant determinant of infection prevention practices during female sterilization (AOR: 2.42; 95% CI: 1.77**–**3.32; Table 4). Moreover, adherence to infection prevention practices was significantly higher in Bihar’s public health facilities than in all UP’s facilities. Availability of staff trained in infection prevention or female sterilization had no significant influence on adherence to infection prevention practice, suggesting the importance of not only training for performance of female sterilization but in infection prevention preparedness.

**Table 4:** Adjusted odds ratio (95% of confidence interval) based on binary logistic regression analysis showing determinants of infection prevention practices adopted during female sterilization in Bihar and Uttar Pradesh

	Adjusted odds ratio
All four infection prevention components available	
*No (Ref)*	
Yes	**2.42 (1.77–3.32)**
Type of facility	
UP private *(Ref)*	
UP public	0.86 (0.48–1.57)
Bihar public	**2.16 (1.12–4.17)**
Facilities with a staff responsible for sterilizing the equipment is trained in infection prevention	
No *(Ref)*	
Yes	0.93 (0.67–1.30)
Facilities with at least one trained provider in female sterilization	
No *(Ref)*	
Yes	0.90 (0.64–1.28)

Ref: Reference category

Adjusted odds ratio in bold are significant at *p* < 0.05

## Discussion

Findings of this study indicate that a considerable proportion of health facilities in Bihar and Uttar Pradesh were not fully adhering to infection prevention practices, even when the facilities have preparedness for infection prevention. Facility readiness and infection prevention practices varied by state. In Bihar, adherence to infection prevention practices were much better in facilities that were fully prepared. But in Uttar Pradesh, facility preparedness was generally low, and providers did not adhere to infection prevention practices even in the facilities that had full preparedness. Poor infection prevention practices in Uttar Pradesh—even when facilities had equipment and supplies —reflect provider lack of adherence to protocols/guidelines. The reasons for such practice may be many. Few studies indicate that poor compliance to infection prevention practices when facilities are prepared may be the result of either of the following reasons: lack of adequate training and experience, excessive workloads, lack of staffs, lack of standard procedures ensuring protocol adherence, and lack of priority for hand hygiene [36, 37].

Inadequacy of mechanisms for equipment sterilization contributes to lower level of protocol adherence. Provision of family planning methods on fixed (one or two) days of the week attracts a large number of clients seeking those services, not allowing enough time to sterilize the equipment even if mechanisms for sterilization exist. Hence, low use of sterilized equipment could be the result of inadequate supplies, high workloads, and insufficient human resources, which have also been identified as factors responsible for poor adherence to infection prevention, in an earlier study based on a literature review [38].

Availability of surgical gloves was almost universal in all three sectors’ health facilities. This finding is similar to studies in Uganda that reported adequate surgical glove availability, in comparison to other infection prevention components [39, 40]. Near universal availability of gloves is likely due to their relative affordability compared to personal protective gear such as gowns. High level of handwashing and gloves use during procedures revealed in this study are comparable with studies in Saudi Arabia and Switzerland [41–43]. Adherence to hand washing does not require expensive resources, and adequate availability of both water and surgical gloves are probable reasons for near-universal adherence to these practices.

Despite government guidelines for quality clinical family planning services, infection prevention practices were not universally adhered during female sterilization procedures in all three health sectors of the study states. Unsterilized equipment used during procedures can cause infection, which may lead to morbidity and mortality, as infections have been leading cause of female sterilization-attributable deaths in several countries [21]. Infection and sepsis lead to hospital visits and hospitalization, increasing both expenditures for care and social suffering by families, as evident in previous studies [44, 45]. In addition to individual and familial consequences, inadequate infection prevention practices will affect India’s family planning program in general due to low satisfaction among women, leading to reduced service uptake. Previous studies from developing countries show low satisfaction followed by discontinuation of family planning services when they are of poor quality [46, 47].

Given that female sterilization is the primary family planning method in majority of states in India, the findings of this study suggest that facility readiness for infection prevention and adherence of the practice are important for improved quality of family planning services. Availability/provision of sterile equipment was the lowest among four components of the infection prevention readiness, which further suggests that facilities should have the required equipment and a dedicated person on staff trained to sterilize instruments. Staffing levels could be ameliorated by central sterilization centers, possibly at district headquarters. The findings further reveal that infection prevention practices are far from universal, even when required commodities and equipment are available. Providers must be trained to focus on adherence to infection prevention practices, with quality control checks for female sterilization. Quality of services should be a high priority of India’s family planning program, to achieve its Sustainable Development Goal of universal access to health care including family planning services by 2030.

Although these findings offer important insights on facility readiness and practices for infection prevention among public and private health facilities in Bihar and Uttar Pradesh, results must be interpreted within the context of certain study limitations. First, the study data are from a baseline evaluation of an ongoing program of state technical support by professional non-governmental organizations for improving quality of care in family planning and maternal and newborn health services. Technical support in Bihar, for example, includes a dedicated individual for each block’s public health facilities to ensure essential components for infection prevention in the operation theatre. This investment in independent block staff has ensured universal facility readiness for infection prevention. Second, the study did not capture contextual factors that may lead to lower levels of infection prevention practice, for example, high client loads, lack of staff motivation, or inadequate number of trained personnel. Previous studies indicate these are primary reasons for insufficient infection prevention practice. Third, there was a slight difference in observation methodology between Bihar and Uttar Pradesh, which may have affected the higher levels of infection prevention practices in Bihar than in Uttar Pradesh.

## Conclusions

The findings emerged with several key messages related to infection prevention preparedness and practices in north India. *First*, availability of staff trained in infection prevention for equipment sterilization was a key challenge in all of the three health sectors, and mechanisms for equipment sterilization were more of a challenge in UP’s public and private facilities; *second*, adherence to all four infection practices during clinical procedures was not universal; *third*, adherence to infection prevention practices is higher during initial cases in facilities prepared for infection prevention, suggesting the importance of client load in influencing practice. Strengthening facilities prepared for infection prevention in proportion to their client loads and changing provider behaviors in relation to infection prevention practice will lead to higher levels of client satisfaction, continuation of contraceptive use, and reductions in out-of-pocket expenditures for the care of complications due to the procedure, if any. Strengthening quality assurance monitoring from local and national committees can also improve adherence to infection prevention protocols during sterilization procedures, and hence overall quality of care in family planning service provision.

## Data Availability

Data and study materials (checklist of facility observation and questionnaire) would be made available by the corresponding author upon reasonable request. However, part of the data related to this study is already published elsewehere. Please see reference number 34.

## Abbreviations

CC: Correlation coefficient
CHC: Community Health Center
CI: Confidence interval
DH: District Hospital
HLD: High level disinfectant
HLFPPT: Hindustan Latex Family Planning Trust
AOR: Adjusted odds ratio
PHC: Primary Health Centers
PPS: Probability proportional to size
PSI: Population Services International
TSU: Technical Support Unit
UP: Uttar Pradesh
